# Crystal structure of di-μ-chlorido-bis(chlorido­{*N* ^1^-phenyl-*N* ^4^-[(pyridin-2-yl-κ*N*)methyl­idene]benzene-1,4-di­amine-κ*N* ^4^}mercury(II))

**DOI:** 10.1107/S2056989015015790

**Published:** 2015-08-29

**Authors:** Md. Serajul Haque Faizi, Elena V. Prisyazhnaya

**Affiliations:** a Department of Chemistry, College of Science, Sultan Qaboos University, P O Box 36 Al-Khod 123, Muscat, Sultanate of Oman; b Department of Chemistry, Kyiv National University of Construction and Architecture, Povitroflotsky Avenue 31, 03680 Kiev, Ukraine

**Keywords:** crystal structure, mercury(II), Schiff base, bidentate ligand, inversion symmetry, hydrogen bonding

## Abstract

The whole mol­ecule of the title complex, [Hg_2_Cl_4_(C_18_H_15_N_3_)_2_], is generated by inversion symmetry. It was synthesized from the pyridine-derived Schiff base *N*-phenyl-*N*′-[(pyridin-2-yl)methyl­idene]benzene-1,4-di­amine (PPMBD). The five-coordinated Hg^2+^ ions have a distorted square-pyramidal environment defined by two N atoms, *viz.* the imine and the other pyridyl [Hg—N = 2.467 (6) and 2.310 (6) Å, respectively] belonging to the bidentate imino­pyridine ligand, and three Cl atoms [Hg—Cl = 2.407 (2), 2.447 (2) and 3.031 (2) Å]. The longest Hg—Cl bond is bridging about the inversion centre. In the ligand, the central ring and pyridine ring are oriented at a dihedral angle of 8.1 (4)°, while the planes of the pyridine ring and the terminal phenyl ring are oriented at a dihedral angle of 53.8 (4)°. In the crystal, mol­ecules are linked by N—H⋯Cl and C—H⋯Cl hydrogen bonds, forming sheets parallel to (001).

## Related literature   

For applications of pyridincarbaldehyde and related structures, see: Baul *et al.* (2004 ▸); Das *et al.* (2013 ▸); Faizi & Sen (2014 ▸); Hughes & Prince (1978 ▸); Jursic *et al.* (2002 ▸); Kasselouri *et al.* (1993 ▸); Mandal *et al.* (2012 ▸); Motswainyana *et al.* (2013 ▸); Song *et al.* (2011 ▸).
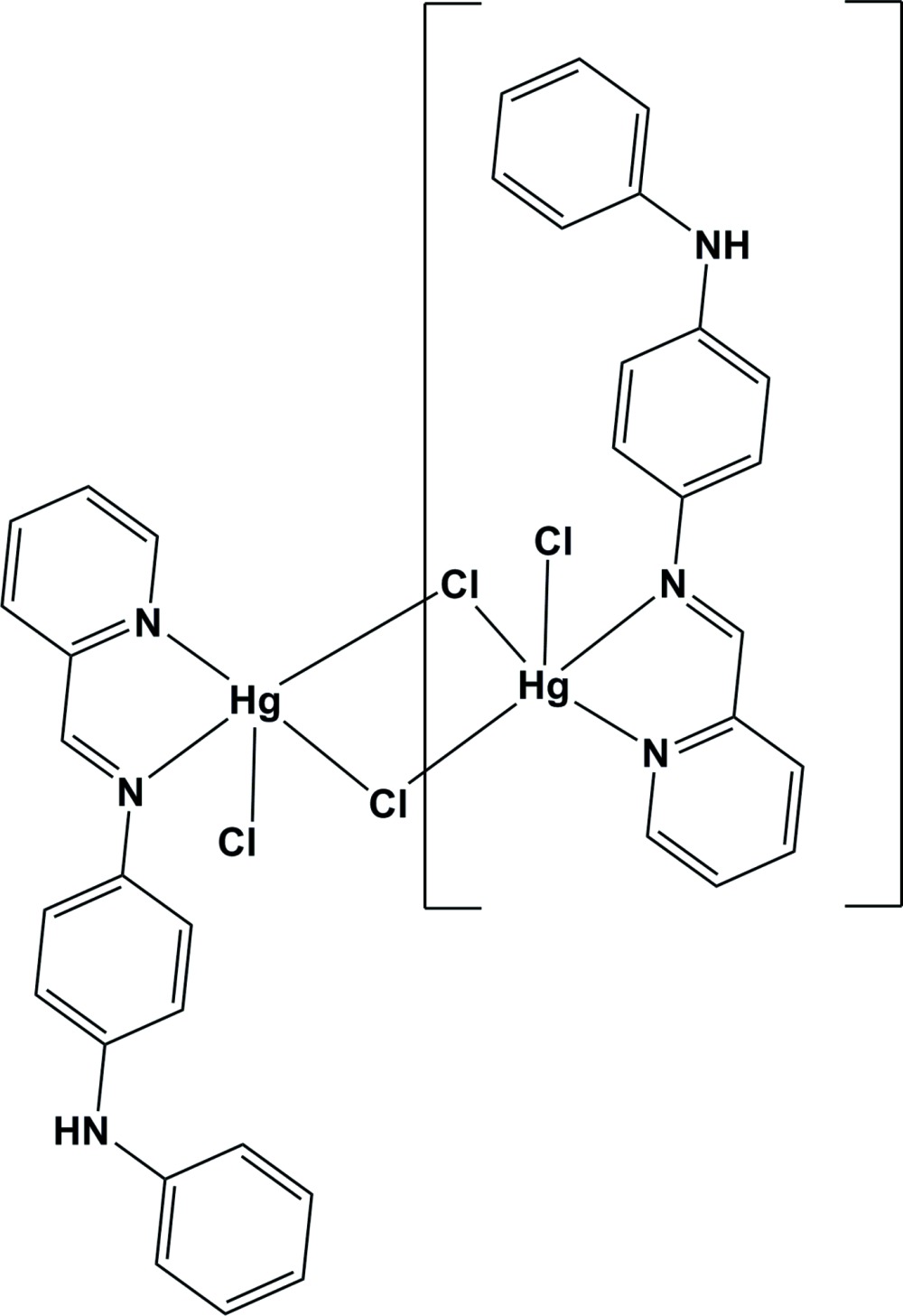


## Experimental   

### Crystal data  

[Hg_2_Cl_4_(C_18_H_15_N_3_)_2_]
*M* *_r_* = 1089.64
Monoclinic, 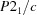

*a* = 11.7507 (14) Å
*b* = 8.9026 (11) Å
*c* = 17.050 (2) Å
β = 90.194 (8)°
*V* = 1783.6 (4) Å^3^
*Z* = 2
Mo *K*α radiation
μ = 8.93 mm^−1^
*T* = 100 K
0.18 × 0.15 × 0.12 mm

### Data collection  

Bruker SMART APEX CCD diffractometer
Absorption correction: multi-scan (*SADABS*; Sheldrick, 2004 ▸) *T*_min_ = 0.296, *T*_max_ = 0.414
19428 measured reflections
4451 independent reflections
2451 reflections with *I* > 2σ(*I*)
*R*_int_ = 0.098

### Refinement  

*R*[*F* ^2^ > 2σ(*F* ^2^)] = 0.048
*wR*(*F* ^2^) = 0.124
*S* = 0.96
4451 reflections
220 parameters
1 restraint
H-atom parameters constrained
Δρ_max_ = 1.78 e Å^−3^
Δρ_min_ = −1.13 e Å^−3^

### 

Data collection: *SMART* (Bruker, 2003 ▸); cell refinement: *SAINT* (Bruker, 2003 ▸); data reduction: *SAINT*; program(s) used to solve structure: *SIR97* (Altomare *et al.*, 1999 ▸); program(s) used to refine structure: *SHELXL2014* (Sheldrick, 2015 ▸); molecular graphics: *Mercury* (Macrae *et al.*, 2008 ▸); software used to prepare material for publication: *SHELXL2014* and *PLATON* (Spek, 2009 ▸).

## Supplementary Material

Crystal structure: contains datablock(s) global, I. DOI: 10.1107/S2056989015015790/su5192sup1.cif

**Fig. 1.**
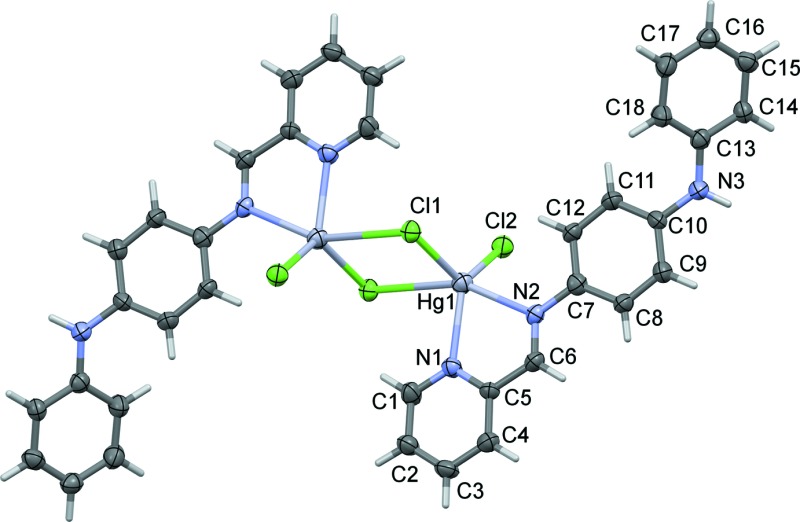
The molecular structure of the title compound, with atom labelling. Displacement ellipsoids are drawn at the 50% probability level. The unlabelled atoms are related to the labelled atoms by inversion symmetry (symmetry code: -x+1, -y+1, -z+1).

**Fig. 2.**
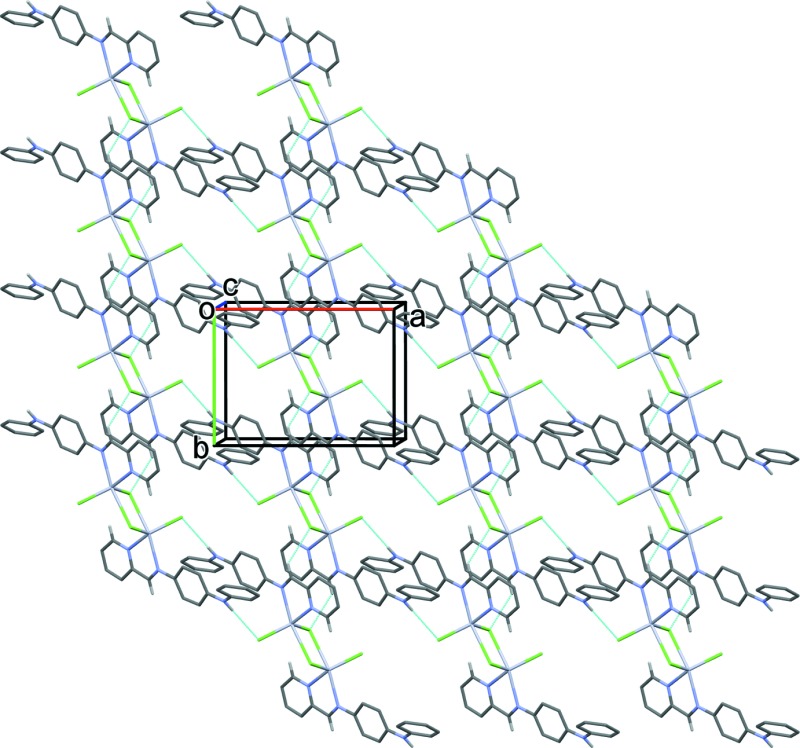
The crystal packing of the title compound viewed along the c axis. The hydrogen bonds are shown as dashed lines (see Table 1 for details), and for clarity only the H atoms involved in hydrogen bonding are shown.

Structure factors: contains datablock(s) I. DOI: 10.1107/S2056989015015790/su5192Isup2.hkl

x y z . DOI: 10.1107/S2056989015015790/su5192fig1.tif
The mol­ecular structure of the title compound, with atom labelling. Displacement ellipsoids are drawn at the 50% probability level. The unlabelled atoms are related to the labelled atoms by inversion symmetry (symmetry code: −*x* + 1, −*y* + 1, −*z* + 1).

c . DOI: 10.1107/S2056989015015790/su5192fig2.tif
The crystal packing of the title compound viewed along the *c* axis. The hydrogen bonds are shown as dashed lines (see Table 1 for details), and for clarity only the H atoms involved in hydrogen bonding are shown.

CCDC reference: 1420119

Additional supporting information:  crystallographic information; 3D view; checkCIF report

## Figures and Tables

**Table 1:** Hydrogen-bond geometry (, )

*D*H*A*	*D*H	H*A*	*D* *A*	*D*H*A*
N3H3*N*Cl2^i^	0.87(2)	2.67(3)	3.510(7)	161(7)
C1H1Cl1^ii^	0.95	2.74	3.493(9)	136
C6H6Cl1^iii^	0.95	2.82	3.526(9)	132

Symmetry codes: (i) 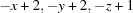; (ii) 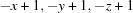; (iii) 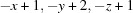.

## supplementary crystallographic information

### S1. Comment

Mercury is one of the most prevalent toxic metals in the environment and gains access to the body orally or dermally, causing cell dysfunction that consequently leads to health problems (Mandal *et al.*, 2012). Schiff base complexes of 2-pyridine­carboxaldehyde and its derivatives have been found to be good herbicides and used for the protection of plants (Hughes & Prince, 1978). Transition metal complexes of pyridyl Schiff bases have found applications in catalysis (Kasselouri *et al.*, 1993), Pyridyl derivatives of Schiff bases are important building blocks of many important compounds widely used in biological applications such as anti­oxidative,anti­cancer, fluorescent probe agents in industry, in coordination chemistry and in catalysis (Motswainyana *et al.*, 2013; Das *et al.* , 2013; Song *et al.* 2011; Jursic *et al.*, 2002). The synthesis of a complex of mercury(II) using the 2-pyridincarbaldehyde derivative of the Schiff base *N*-phenyl-*N*'-pyridin-2-yl­methyl­ene benzene-1,4-di­amine (PPMBD) has not previously been reported. We report herein the crystal structure of a new mercury(II) complex of this ligand.

The whole molecule of the title complex, Fig. 1, is generated by inversion symmetry. The Schiff base derived PPMBD ligand coordinates to the Hg^II^ atom as a bidentate ligand through the N atoms of the imine group and pyridine ring. Also two bridging and one terminal chloride anions are present in the coordination environment of the Hg^II^ atom (Baul *et al.*, 2004). The five-coordinated Hg^2+^ ions have a distorted square-pyramidal geometry defined by two N atoms viz. one imine, the other pyridyl [Hg–N = 2.467 (6) and 2.310 (6) Å, respectively], belonging to the bidentate imino­pyridine ligand and three Cl atoms [Hg—Cl = 2.407 (2), 2.447 (2) and 3.031 (2) Å]. The longest Hg—Cl distance, Hg1···Cl1^i^ = 3.031 (2) Å, is bridging about the centre of inversion (symmetry code: (i) -x+1, -y+1, -z+1). The observed Hg—Cl and Hg—N bond lengths and bond angles are considered normal for this type of Hg^II^ complex (Faizi & Sen, 2014). The central ring and pyridine ring are oriented at a dihedral angle of 8.10 (6)°. The pyridine ring and terminal phenyl ring are oriented at a dihedral angle of 53.78 (6)°.

In the crystal, molecules are linked by N—H···Cl and C—H···Cl hydrogen bonds forming sheets parallel to (001); see Fig. 2 and Table 1.

### S2. Synthesis and crystallization

The imino­pyridyl compound *N*-phenyl-*N*'-pyridin-2-yl­methyl­ene benzene-1,4-di­amine (PPMBD) was prepared by adding drop wise pyridine-2-carbaldehyde (0.29 g, 2.71 mmol) to a methano­lic solution (50 ml) of *N*-phenyl-*p*-phenyl­enedi­amine (0.50 g, 2.71 mmol). The reaction mixture was stirred for 3 h at room temperature and filtered. The resulting yellow solid powder was washed with methanol (2 × 3 ml) and hexane (3 × 10 ml), respectively. The compound was recrystallized from in hot MeOH to give yellow crystals, which were dried in a vacuum desiccator to give the pure product (yield: 0.60 g, 80%; m.p.: 410-142 K). UV/vis (MeOH): λmax, nm (ε, M^-1^ cm^-1^): 205 (40,000), 280 (18,000), 398 (18,000). IR (KBr, cm^-1^): ν(N—H) 3259, ν(HC=N) 1618. ^1^H NMR (400 MHz DMSO-d_6_) δ (ppm) 8.67 (^1^H, d, J = 4.8 Hz), 8.41 (^1^H, s, HC=N), 8.12 (^1^H, d, J = 4.4 Hz), 7.90 (^1^H, t, J = 8.0 Hz), 7.46 (^1^H, t, J = 7.6 Hz ) 7.35 (^2^H, d, J = 3.6 Hz), 7.25 (^2^H, t, J = 3.6 Hz), 7.2 (^2^H, m, J = 7.2), 7.12 (^2^H, m), 6.86 (^1^H, t). HRMS (ESI) m/z [M+H]+ calcd for C_18_H_15_N_3_: 274.1339 found: 274.1349.

The title compound was prepared by reacting (PPMBD) (0.100 g, 0.37 mmol) with mercury(II) chloride (0.099 g, 0.37 mmol) in methanol (5 ml), with vigorous stirring for 2 h at room temperature The yellow precipitate that formed was filtered off and redissolved in di­methyl­formamide. Crystals of the title complex suitable for X-ray analysis was obtained within 3 days by slow evaporation of the di­methyl­formamide. The yellow crystals of the title compound were isolated (yield: 0.31 g, 77.1%; m.p.: 520 K).

### S3. Refinement

Crystal data, data collection and structure refinement details are summarized in Table 2. The NH H-atom was located in difference Fourier map and refined with a distance restraint: N—H = 0.88 (2) Å with *U*_iso_(H) = 1.2*U*_eq_(N). The C-bound H-atoms were positioned geometrically and refined using a riding model: C—H = 0.95 Å with *U*_iso_(H) = 1.2*U*_eq_(C).

### Crystal data

**Table:**

[Hg_2_Cl_4_(C_18_H_15_N_3_)_2_]	*Z* = 2
*M**_r_* = 1089.64	*F*(000) = 1032
Monoclinic, *P*2_1_/*c*	*D*_x_ = 2.029 Mg m^−^^3^
*a* = 11.7507 (14) Å	Mo *K*α radiation, λ = 0.71073 Å
*b* = 8.9026 (11) Å	µ = 8.93 mm^−^^1^
*c* = 17.050 (2) Å	*T* = 100 K
β = 90.194 (8)°	Needle, yellow
*V* = 1783.6 (4) Å^3^	0.18 × 0.15 × 0.12 mm

### Data collection

**Table:**

Bruker SMART APEX CCD diffractometer	4451 independent reflections
Radiation source: fine-focus sealed tube	2451 reflections with *I* > 2σ(*I*)
Graphite monochromator	*R*_int_ = 0.098
/w–scans	θ_max_ = 28.5°, θ_min_ = 2.4°
Absorption correction: multi-scan (*SADABS*; Sheldrick, 2004)	*h* = −15→15
*T*_min_ = 0.296, *T*_max_ = 0.414	*k* = −11→11
19428 measured reflections	*l* = −22→22

### Refinement

**Table:**

Refinement on *F*^2^	1 restraint
Least-squares matrix: full	Hydrogen site location: mixed
*R*[*F*^2^ > 2σ(*F*^2^)] = 0.048	H-atom parameters constrained
*wR*(*F*^2^) = 0.124	*w* = 1/[σ^2^(*F*_o_^2^) + (0.0446*P*)^2^] where *P* = (*F*_o_^2^ + 2*F*_c_^2^)/3
*S* = 0.96	(Δ/σ)_max_ = 0.001
4451 reflections	Δρ_max_ = 1.78 e Å^−^^3^
220 parameters	Δρ_min_ = −1.13 e Å^−^^3^

### Special details

**Table:**

Geometry. All esds (except the esd in the dihedral angle between two l.s. planes) are estimated using the full covariance matrix. The cell esds are taken into account individually in the estimation of esds in distances, angles and torsion angles; correlations between esds in cell parameters are only used when they are defined by crystal symmetry. An approximate (isotropic) treatment of cell esds is used for estimating esds involving l.s. planes.

### Fractional atomic coordinates and isotropic or equivalent isotropic displacement parameters (Å^2^)

**Table:**

	*x*	*y*	*z*	*U*_iso_*/*U*_eq_	
Hg1	0.60187 (3)	0.67045 (4)	0.53562 (2)	0.06131 (16)	
Cl1	0.50851 (17)	0.6189 (2)	0.40995 (11)	0.0574 (5)	
Cl2	0.78086 (16)	0.5540 (2)	0.56798 (12)	0.0631 (5)	
N1	0.4820 (5)	0.7921 (7)	0.6219 (4)	0.0525 (16)	
N2	0.6534 (5)	0.9389 (7)	0.5409 (3)	0.0474 (15)	
N3	1.0303 (6)	1.1588 (8)	0.3773 (4)	0.0633 (19)	
H3N	1.072 (6)	1.223 (8)	0.403 (4)	0.076*	
C1	0.3949 (7)	0.7282 (10)	0.6584 (5)	0.068 (2)	
H1	0.3772	0.6263	0.6471	0.082*	
C2	0.3290 (7)	0.8051 (10)	0.7125 (5)	0.066 (2)	
H2	0.2672	0.7562	0.7375	0.079*	
C3	0.3529 (7)	0.9481 (11)	0.7291 (5)	0.065 (2)	
H3	0.3106	1.0011	0.7677	0.078*	
C4	0.4402 (7)	1.0173 (11)	0.6891 (4)	0.064 (2)	
H4	0.4569	1.1203	0.6983	0.076*	
C5	0.5036 (6)	0.9358 (9)	0.6355 (4)	0.0473 (18)	
C6	0.5981 (6)	1.0109 (10)	0.5924 (4)	0.057 (2)	
H6	0.6168	1.1125	0.6035	0.069*	
C7	0.7473 (6)	0.9994 (10)	0.4998 (4)	0.0543 (19)	
C8	0.7938 (7)	1.1435 (9)	0.5152 (5)	0.060 (2)	
H8	0.7601	1.2064	0.5537	0.073*	
C9	0.8865 (7)	1.1914 (10)	0.4749 (4)	0.063 (2)	
H9	0.9190	1.2863	0.4870	0.076*	
C10	0.9349 (6)	1.1035 (10)	0.4159 (4)	0.054 (2)	
C11	0.8901 (7)	0.9631 (10)	0.3999 (4)	0.057 (2)	
H11	0.9231	0.9016	0.3605	0.069*	
C12	0.7970 (7)	0.9128 (9)	0.4417 (4)	0.055 (2)	
H12	0.7663	0.8164	0.4302	0.066*	
C13	1.0727 (7)	1.1208 (9)	0.3032 (4)	0.055 (2)	
C14	1.1864 (7)	1.1437 (9)	0.2874 (5)	0.056 (2)	
H14	1.2346	1.1839	0.3270	0.067*	
C15	1.2311 (7)	1.1094 (10)	0.2154 (5)	0.064 (2)	
H15	1.3101	1.1235	0.2064	0.077*	
C16	1.1642 (8)	1.0554 (10)	0.1565 (5)	0.064 (2)	
H16	1.1957	1.0316	0.1068	0.077*	
C17	1.0504 (8)	1.0362 (11)	0.1704 (5)	0.073 (3)	
H17	1.0030	0.9989	0.1296	0.088*	
C18	1.0023 (7)	1.0698 (10)	0.2428 (4)	0.067 (3)	
H18	0.9228	1.0583	0.2510	0.080*	

### Atomic displacement parameters (Å^2^)

**Table:**

	*U*^11^	*U*^22^	*U*^33^	*U*^12^	*U*^13^	*U*^23^
Hg1	0.0548 (2)	0.0531 (2)	0.0761 (3)	−0.00007 (16)	0.00288 (16)	−0.01027 (17)
Cl1	0.0604 (13)	0.0501 (12)	0.0617 (11)	−0.0058 (10)	0.0020 (9)	0.0005 (10)
Cl2	0.0529 (12)	0.0545 (13)	0.0818 (13)	0.0041 (10)	−0.0016 (10)	−0.0070 (11)
N1	0.049 (4)	0.046 (4)	0.062 (4)	0.002 (3)	0.008 (3)	0.000 (3)
N2	0.053 (4)	0.038 (4)	0.051 (3)	−0.001 (3)	−0.005 (3)	0.000 (3)
N3	0.063 (5)	0.069 (5)	0.058 (4)	−0.017 (4)	0.012 (3)	−0.017 (4)
C1	0.059 (6)	0.047 (5)	0.098 (6)	−0.002 (4)	0.012 (5)	−0.002 (5)
C2	0.059 (6)	0.062 (7)	0.076 (6)	0.002 (4)	0.021 (4)	−0.003 (5)
C3	0.064 (6)	0.064 (6)	0.068 (5)	0.010 (5)	0.024 (4)	−0.006 (5)
C4	0.064 (6)	0.059 (6)	0.068 (5)	0.000 (4)	0.004 (4)	−0.016 (5)
C5	0.043 (4)	0.046 (5)	0.054 (4)	0.004 (3)	0.006 (3)	−0.007 (4)
C6	0.064 (5)	0.045 (5)	0.062 (5)	−0.004 (4)	0.006 (4)	−0.011 (4)
C7	0.058 (5)	0.047 (5)	0.057 (4)	0.000 (4)	0.001 (4)	0.008 (4)
C8	0.070 (6)	0.050 (6)	0.062 (5)	−0.010 (4)	0.015 (4)	−0.013 (4)
C9	0.060 (6)	0.063 (7)	0.067 (5)	−0.015 (4)	0.012 (4)	−0.008 (4)
C10	0.044 (5)	0.065 (6)	0.052 (4)	−0.006 (4)	0.010 (4)	−0.008 (4)
C11	0.059 (5)	0.050 (5)	0.062 (5)	0.001 (4)	0.008 (4)	−0.012 (4)
C12	0.064 (5)	0.040 (5)	0.061 (5)	−0.004 (4)	0.006 (4)	−0.011 (4)
C13	0.057 (5)	0.046 (5)	0.060 (5)	−0.001 (4)	0.001 (4)	0.000 (4)
C14	0.046 (5)	0.055 (5)	0.066 (5)	−0.006 (4)	0.001 (4)	0.003 (4)
C15	0.061 (6)	0.059 (6)	0.073 (6)	0.008 (4)	0.014 (5)	−0.001 (5)
C16	0.080 (6)	0.054 (6)	0.058 (5)	−0.005 (5)	0.011 (4)	−0.002 (4)
C17	0.075 (6)	0.074 (7)	0.070 (5)	−0.017 (5)	−0.007 (5)	−0.006 (5)
C18	0.053 (5)	0.087 (8)	0.062 (5)	−0.011 (5)	−0.001 (4)	−0.009 (5)

### Geometric parameters (Å, º)

**Table:**

Hg1—N1	2.310 (6)	C7—C12	1.388 (10)
Hg1—Cl2	2.407 (2)	C7—C8	1.418 (11)
Hg1—Cl1	2.4474 (19)	C8—C9	1.360 (10)
Hg1—N2	2.467 (6)	C8—H8	0.9500
N1—C5	1.325 (9)	C9—C10	1.396 (10)
N1—C1	1.327 (10)	C9—H9	0.9500
N2—C6	1.269 (9)	C10—C11	1.383 (11)
N2—C7	1.414 (9)	C11—C12	1.381 (10)
N3—C10	1.392 (10)	C11—H11	0.9500
N3—C13	1.401 (9)	C12—H12	0.9500
N3—H3N	0.87 (2)	C13—C14	1.379 (10)
C1—C2	1.387 (11)	C13—C18	1.394 (10)
C1—H1	0.9500	C14—C15	1.371 (10)
C2—C3	1.334 (11)	C14—H14	0.9500
C2—H2	0.9500	C15—C16	1.360 (11)
C3—C4	1.380 (11)	C15—H15	0.9500
C3—H3	0.9500	C16—C17	1.370 (11)
C4—C5	1.386 (10)	C16—H16	0.9500
C4—H4	0.9500	C17—C18	1.392 (10)
C5—C6	1.491 (10)	C17—H17	0.9500
C6—H6	0.9500	C18—H18	0.9500
			
N1—Hg1—Cl2	126.16 (16)	N2—C7—C8	123.6 (7)
N1—Hg1—Cl1	111.91 (16)	C9—C8—C7	120.0 (8)
Cl2—Hg1—Cl1	120.57 (7)	C9—C8—H8	120.0
N1—Hg1—N2	70.9 (2)	C7—C8—H8	120.0
Cl2—Hg1—N2	101.23 (15)	C8—C9—C10	121.2 (8)
Cl1—Hg1—N2	108.84 (13)	C8—C9—H9	119.4
C5—N1—C1	118.7 (7)	C10—C9—H9	119.4
C5—N1—Hg1	116.5 (5)	C11—C10—N3	122.2 (7)
C1—N1—Hg1	124.8 (6)	C11—C10—C9	119.5 (8)
C6—N2—C7	123.5 (7)	N3—C10—C9	118.2 (8)
C6—N2—Hg1	112.8 (5)	C12—C11—C10	119.5 (7)
C7—N2—Hg1	122.9 (5)	C12—C11—H11	120.2
C10—N3—C13	129.0 (7)	C10—C11—H11	120.2
C10—N3—H3N	117 (5)	C11—C12—C7	121.6 (7)
C13—N3—H3N	114 (6)	C11—C12—H12	119.2
N1—C1—C2	122.2 (8)	C7—C12—H12	119.2
N1—C1—H1	118.9	C14—C13—C18	118.4 (7)
C2—C1—H1	118.9	C14—C13—N3	119.2 (7)
C3—C2—C1	119.7 (8)	C18—C13—N3	122.2 (7)
C3—C2—H2	120.1	C15—C14—C13	121.1 (7)
C1—C2—H2	120.1	C15—C14—H14	119.4
C2—C3—C4	118.5 (8)	C13—C14—H14	119.4
C2—C3—H3	120.8	C16—C15—C14	121.1 (8)
C4—C3—H3	120.8	C16—C15—H15	119.4
C3—C4—C5	119.7 (8)	C14—C15—H15	119.4
C3—C4—H4	120.2	C15—C16—C17	118.6 (8)
C5—C4—H4	120.2	C15—C16—H16	120.7
N1—C5—C4	121.1 (7)	C17—C16—H16	120.7
N1—C5—C6	119.3 (7)	C16—C17—C18	121.8 (8)
C4—C5—C6	119.5 (7)	C16—C17—H17	119.1
N2—C6—C5	119.9 (7)	C18—C17—H17	119.1
N2—C6—H6	120.0	C17—C18—C13	118.9 (8)
C5—C6—H6	120.0	C17—C18—H18	120.6
C12—C7—N2	118.2 (7)	C13—C18—H18	120.6
C12—C7—C8	118.2 (7)		
			
C5—N1—C1—C2	−2.7 (12)	C7—C8—C9—C10	2.6 (13)
Hg1—N1—C1—C2	176.9 (6)	C13—N3—C10—C11	25.0 (14)
N1—C1—C2—C3	−0.1 (13)	C13—N3—C10—C9	−157.8 (8)
C1—C2—C3—C4	2.7 (13)	C8—C9—C10—C11	−2.2 (13)
C2—C3—C4—C5	−2.6 (12)	C8—C9—C10—N3	−179.5 (8)
C1—N1—C5—C4	2.7 (11)	N3—C10—C11—C12	178.2 (7)
Hg1—N1—C5—C4	−176.9 (5)	C9—C10—C11—C12	1.1 (12)
C1—N1—C5—C6	−177.4 (7)	C10—C11—C12—C7	−0.4 (12)
Hg1—N1—C5—C6	2.9 (8)	N2—C7—C12—C11	−178.9 (7)
C3—C4—C5—N1	−0.1 (11)	C8—C7—C12—C11	0.7 (11)
C3—C4—C5—C6	−180.0 (7)	C10—N3—C13—C14	−156.2 (9)
C7—N2—C6—C5	−177.1 (6)	C10—N3—C13—C18	28.2 (14)
Hg1—N2—C6—C5	−6.9 (8)	C18—C13—C14—C15	−3.6 (12)
N1—C5—C6—N2	3.1 (11)	N3—C13—C14—C15	−179.4 (8)
C4—C5—C6—N2	−177.1 (7)	C13—C14—C15—C16	1.8 (13)
C6—N2—C7—C12	−176.5 (7)	C14—C15—C16—C17	0.2 (13)
Hg1—N2—C7—C12	14.2 (9)	C15—C16—C17—C18	−0.2 (14)
C6—N2—C7—C8	4.0 (11)	C16—C17—C18—C13	−1.7 (14)
Hg1—N2—C7—C8	−165.3 (6)	C14—C13—C18—C17	3.5 (13)
C12—C7—C8—C9	−1.8 (12)	N3—C13—C18—C17	179.1 (8)
N2—C7—C8—C9	177.8 (7)		

### Hydrogen-bond geometry (Å, º)

**Table:**

*D*—H···*A*	*D*—H	H···*A*	*D*···*A*	*D*—H···*A*
N3—H3*N*···Cl2^i^	0.87 (2)	2.67 (3)	3.510 (7)	161 (7)
C1—H1···Cl1^ii^	0.95	2.74	3.493 (9)	136
C6—H6···Cl1^iii^	0.95	2.82	3.526 (9)	132

Symmetry codes: (i) −*x*+2, −*y*+2, −*z*+1; (ii) −*x*+1, −*y*+1, −*z*+1; (iii) −*x*+1, −*y*+2, −*z*+1.
