# Supplementary figures and images for: Crystal structure of di-μ-chlorido-bis(chlorido­{N 1-phenyl-N 4-[(pyridin-2-yl-κN)methyl­idene]benzene-1,4-di­amine-κN 4}mercury(II))
Source: Acta Crystallogr E Crystallogr Commun. 2015 Aug 29;71(Pt 9):m175–6. doi: 10.1107/S2056989015015790 (PMC4555402; doi:10.1107/S2056989015015790)

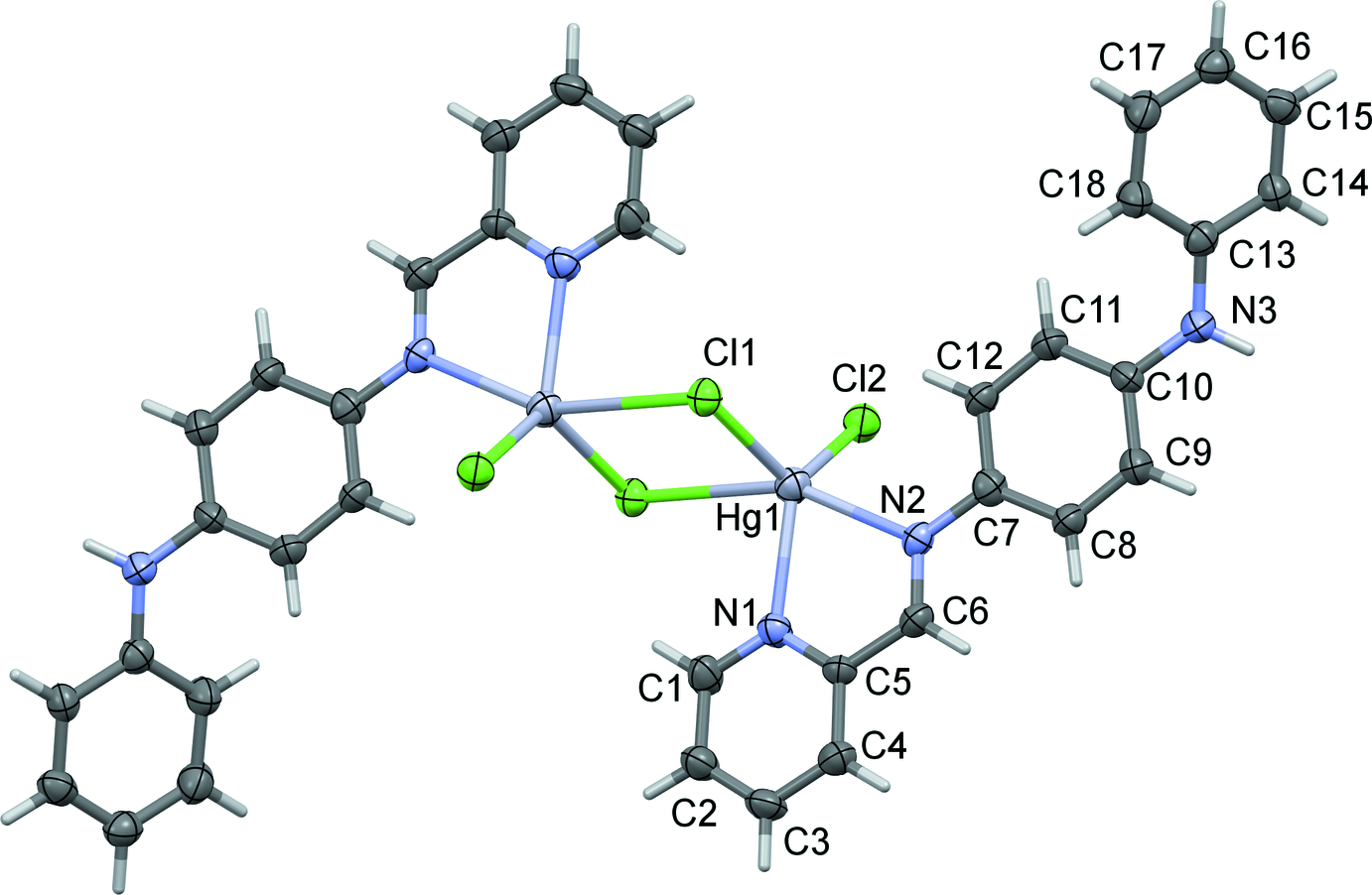

Supplement: Supplementary file 3 [file e-71-0m175-fig1.tif]

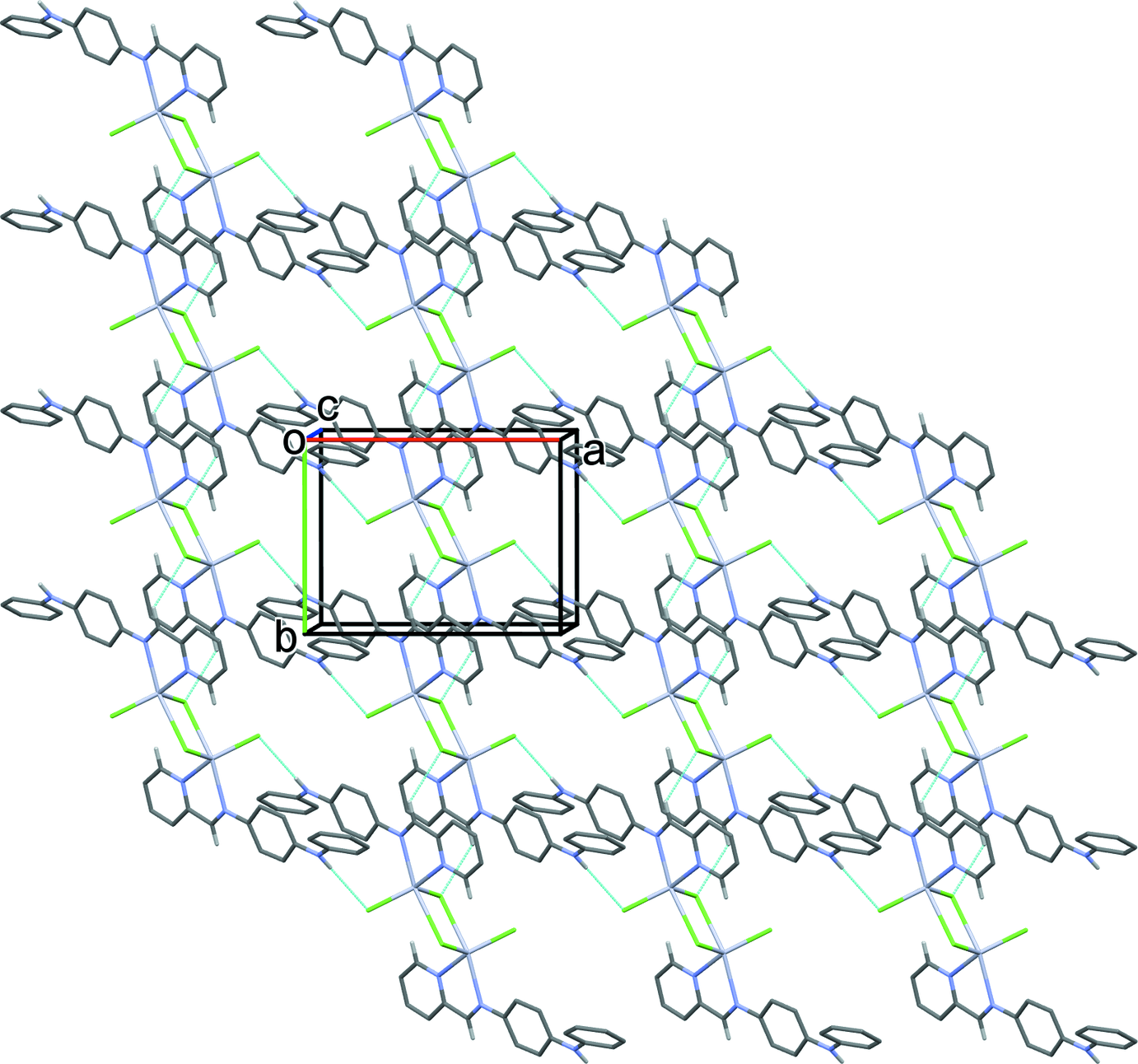

Supplement: Supplementary file 4 [file e-71-0m175-fig2.tif]
